# Challenging Diagnosis of Fahr's Disease Mimicking Parkinson's Disease: A Case Report

**DOI:** 10.1002/ccr3.70250

**Published:** 2025-02-18

**Authors:** Erfan Sanaei, Reza Bidaki, Mohammad Pashmchi, Hamid Jalalifard

**Affiliations:** ^1^ Student Research Committee Shahid Sadoughi University of Medical Sciences Yazd Iran; ^2^ Department of Psychiatry, Research Center of Addiction and Behavioral Sciences, Non‐Communicable Diseases Research Institute Shahid Sadoughi University of Medical Sciences Yazd Iran

**Keywords:** case report, early‐onset Parkinson's disease, Fahr's disease, genetic testing, neuropsychiatric symptoms

## Abstract

Patients presenting with parkinsonism symptoms such as severe bradykinesia and significant hypophonia, along with an atypical clinical course, should be evaluated for secondary parkinsonism syndromes and other causes of parkinsonism. One of these causes is known as Fahr's disease, which is often overlooked by neurologists and other physicians.

**Abbreviations**
CBT: combination behavioral therapy
EOPD: early‐onset Parkinson's disease
FD: Fahr's disease
mg: milligram
PD: Parkinson's disease

## 1 Introduction

Fahr's disease (FD), also known as idiopathic basal ganglia calcification or primary familial brain calcification (PFBC), is a rare and progressive neurological disorder. It is characterized by bilateral and symmetrical calcifications primarily affecting the basal ganglia, the thalamus, cerebellar dentate nuclei, and subcortical white matter [1]. These calcifications can be detected through neuroimaging, such as CT scans and MRIs. Clinically, FD presents with a wide range of neurological and psychiatric symptoms, including movement disorders, cognitive decline, and psychiatric disturbances [2].

The etiology of FD remains not entirely understood, but it is frequently linked to genetic mutations. Mutations in the SLC20A2 gene, which encodes a phosphate transporter, account for about 40% of familial cases [3]. Other implicated genes include PDGFB, PDGFRB, and XPR1, which are involved in cellular phosphate metabolism. These mutations lead to abnormal calcium and phosphate homeostasis, resulting in the pathological calcifications seen in FD [4].

The clinical manifestations of FD are highly variable. Neurological symptoms can include parkinsonism, chorea, dystonia, ataxia, and seizures. Psychiatric manifestations are also common and may include depression, psychosis, and cognitive impairments [5]. The onset of symptoms typically occurs in the fourth to sixth decade of life, although earlier or later presentations can occur [6].

Early‐onset Parkinson's disease (EOPD) is defined by the onset of Parkinsonian symptoms before the age of 50. It shares many clinical features with typical Parkinson's disease (PD), such as bradykinesia, rigidity, tremor, and postural instability [7]. However, EOPD is often distinguished by a stronger genetic component, more frequent dystonia, and a generally more aggressive disease course. Mutations in genes such as PARK2 (parkin), PINK1, and DJ‐1 are commonly associated with EOPD. These genes are involved in mitochondrial function and the ubiquitin‐proteasome system, which are critical for neuronal survival [8].

The misdiagnosis of FD as EOPD in a single patient displays a major clinical challenge. Although both conditions can exhibit similar parkinsonian symptoms like rigidity, bradykinesia, and gait issues, their underlying mechanisms are fundamentally different. FD involves bilateral calcifications in the basal ganglia, thalamus, and cerebellum, whereas EOPD primarily results from the degeneration of dopaminergic neurons in the substantia nigra [9, 10, 11].

This case illustrates the clinical difficulties in distinguishing FD from PD and highlights the importance of a thorough and multidisciplinary approach to accurately diagnose and manage such challenging cases.

## 2 Case History/Examination

A 57‐year‐old Iranian man was referred to our institution's psychiatry clinic. The patient presented with a complex history of progressive neuropsychiatric and motor symptoms. The onset of the patient's symptoms began at the age of 37, classifying this case as an early‐onset variant. The symptoms have progressively worsened over the course of 2 years. Given that the onset occurred before the age of 40, it is categorized as early‐onset. Notably, he reported no substance use, including alcohol or recreational drugs. The patient had a low socioeconomic status and a low body mass index (BMI).

His family history was significant for several neuropsychiatric conditions. His mother had obsessive‐compulsive disorder (OCD), his sister had a history of infantile seizures and borderline intelligence, and an uncle exhibited similar neuropsychiatric symptoms to the patient and died within 6 months of symptom onset. This family history suggested a possible genetic predisposition to neuropsychiatric disorders.

**FIGURE 1:**
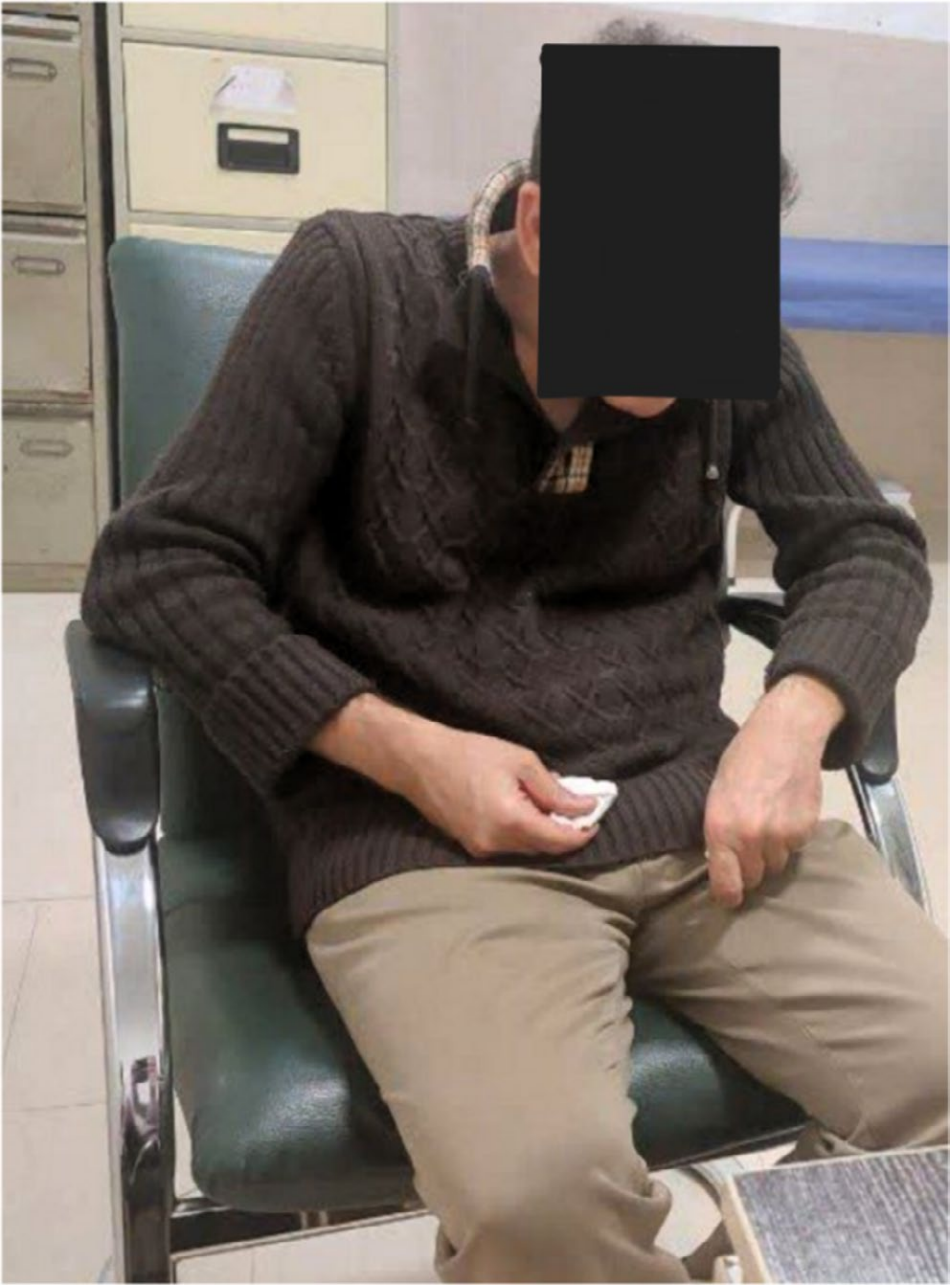
This image shows a Fahr's disease patient with postural instability and abnormal muscle tone. These symptoms are due to brain calcifications characteristic of the disorder.

The patient's initial symptoms included numbness in his right leg and gait disturbances, which progressively worsened. The patient exhibited mild impairment in attention and concentration, while orientation remained intact. Gait was neither spastic nor ataxic but was notably slow, characterized by a shuffling pattern. Additional clinical features included severe motor slowness, significantly impaired gait velocity, and marked bradykinesia. The patient's voice was remarkably subdued, characterized by significantly reduced pitch, resonance, and tonality, creating an impression of a sound emanating from a deep cavity. Over time, the patient also exhibited a range of neuropsychiatric symptoms, including indifference, depressed mood, decreased energy, abulia, anhedonia, paranoid ideation, and psychomotor retardation (Figure 1). During the neuropsychiatric evaluation, the patient exhibited limited cooperation in completing the Montreal Cognitive Assessment (MoCA); however, significant impairments in concentration and reduced cognitive information processing were evident. The cognitive deficit primarily manifested as reduced information processing speed.

In the early stages of the disease, the patient predominantly exhibited psychiatric symptoms, including depression, social withdrawal, and reduced verbal communication. Approximately 36 months later, motor symptoms progressively emerged. This clinical progression suggests that the initial presentation was primarily psychiatric, followed by the development of motor impairments. Additionally, the patient exhibited fatigability, motor clumsiness, and an unsteady gait. These symptoms significantly impacted his quality of life and ability to perform daily activities independently, especially since about 2 years ago.

As his condition deteriorated, he required assistance for basic activities of daily living. He also experienced visual hallucinations, which he described as seeing shadows or figures that were not present. Additionally, he had obsessive thoughts about cleanliness and washing, recognizing these thoughts as irrational but unable to control them. He also reported frequent headaches.

On mental status examination, the patient exhibited poor verbal and eye contact during the mental status examination. His facial expression appeared masked, a common feature in PD, and he maintained a stooped posture with flexed elbows. Due to this circumstance, the patient was misdiagnosed with EOPD and treated with Levodopa. However, the treatment was not entirely effective, and symptoms continued to progress. He appeared semi‐cooperative but had a defensive demeanor. His mood was markedly depressed, and his affect was restricted. His memory was impaired, especially his short‐term recall, and his concentration was notably reduced. Despite these symptoms, he demonstrated partial insight into his condition, acknowledging the abnormality of his experiences but feeling powerless to change them.

This manifestation was characteristic of Parkinsonian syndromes [12], prompting an initial diagnosis and treatment for EOPD. The patient was undergoing treatment with Levodopa and Amantadine. However, over the course of two to three follow‐up visits, no substantial changes in the patient's symptoms were observed. There was only minimal improvement in motor symptoms, and the patient's facial expression remained notably masked. Furthermore, no changes were observed in mood or psychotic symptoms. Antipsychotic treatments were intentionally avoided to prevent potential worsening of the patient's condition.

## 3 Methods (Differential Diagnosis, Investigations, and Treatment)

Neurological examination revealed a shuffling gait, rigidity, reduced arm swing, a positive glabella tap test, and no tremor or dystonia. These findings were consistent with Parkinsonian syndrome but did not entirely explain the full spectrum of his symptoms. Given the complexity of his presentation, a comprehensive diagnostic workup was initiated.

**FIGURE 2:**
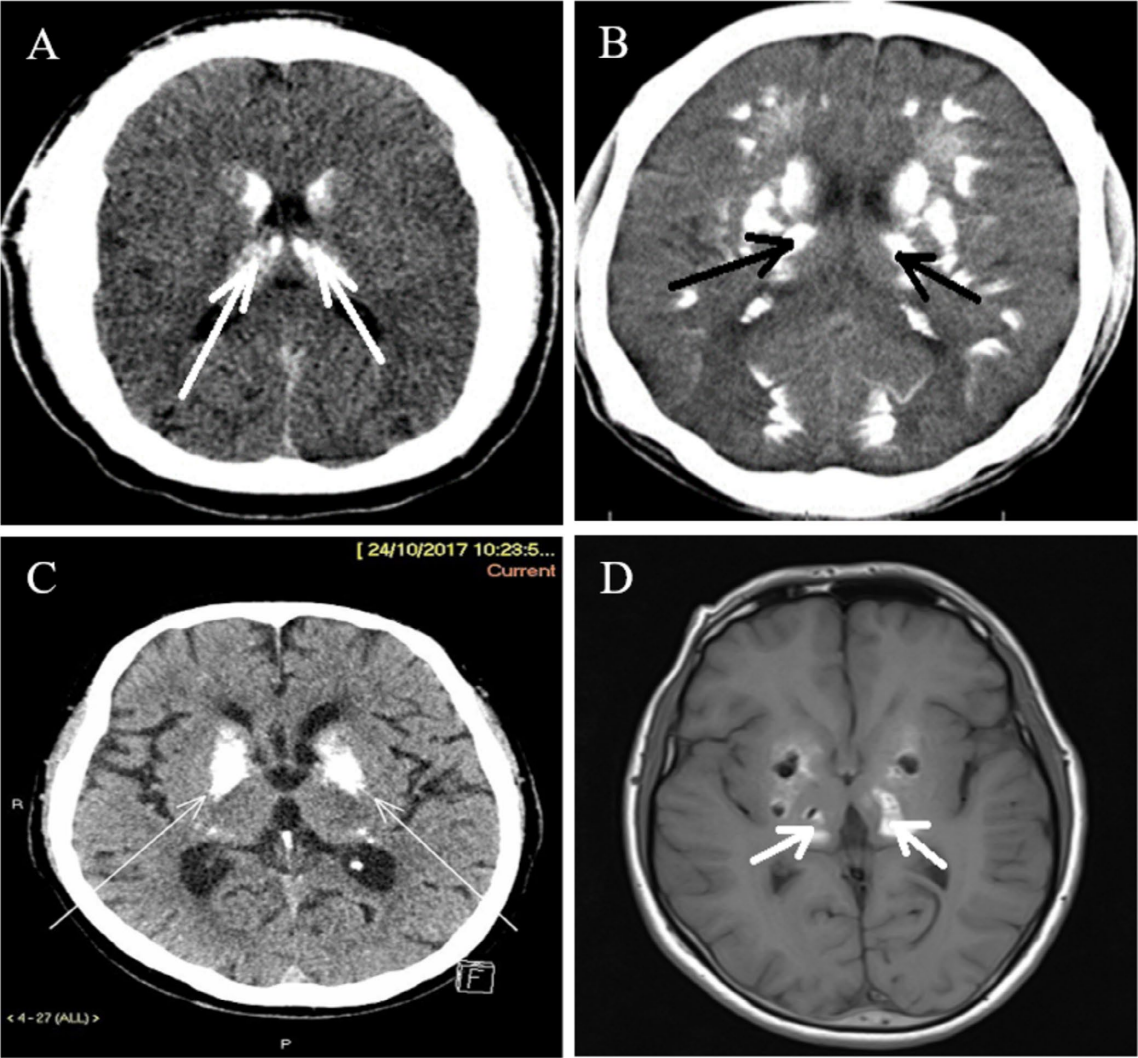
(A, B, C) CT scan of Fahr's disease patient shows bilateral symmetrical calcifications in the basal ganglia and subcortical white matter, characteristic of the disorder. (D) MRI of Fahr's disease patient, highlighting prominent calcium deposits in the basal ganglia, particularly in the globus pallidus and putamen region.

The patient's electroencephalography (EEG) findings were unremarkable and demonstrated normal neural activity. Imaging studies were crucial in identifying the underlying pathology. A computed tomography (CT) scan showed dense calcifications in the basal ganglia, thalamus, and parenchymal regions, which are characteristic of FD. A magnetic resonance imaging (MRI) further revealed symmetrical abnormal signals in the basal ganglia, thalamus, subcortical white matter, and cerebellar hemispheres, suggesting calcification or hemorrhage. Altogether, neuroimaging findings were indicative of FD (Figure 2).

Molecular genetic testing was performed to identify potential genetic causes for his symptoms. The testing identified a homozygous variant in the JAM2 gene, with a possible pathogenic role, and two heterozygous variants in the WNK1 gene, whose pathogenicity was unknown. Further genetic analysis, particularly of the SLC20A2 gene, was recommended to confirm the diagnosis of FD [3].

Based on the clinical presentation, neuroimaging findings, and genetic testing, the patient was re‐diagnosed with FD. However, it was recognized that the initial misdiagnosis of primary PD had led to dopaminergic treatment, which contributed to the development of secondary Parkinsonism due to the underlying basal ganglia calcifications.

The major differential diagnoses for secondary causes of brain calcification, including cysticercosis, lead toxicity, hyperparathyroidism, manganese toxicity, and Wilson's disease, were systematically excluded through detailed clinical history, and multidisciplinary consultations with infectious disease specialists, internists, and neurologists. To rule out the suspected condition, comprehensive laboratory analyses, including a complete blood count (CBC) screening, revealed no abnormalities. Vital organ function tests were within normal limits, and serum levels of calcium, magnesium, and phosphorus were normal. Additionally, infectious disease screenings, including tests for toxoplasmosis, were negative. Collateral information regarding the patient's condition was obtained from the spouse. Additionally, one of our major challenges for diagnosis was the high cost of diagnostic tests, especially genetic evaluations, which caused financial limitations for the patient. The patient was also evaluated for schizophrenia based on DSM‐5 criteria, which did not support the diagnosis. Also, the patients underwent the Movement Disorder Society (MDS) criteria for diagnosing PD and parkinsonism [12].

## 4 Conclusion and Results (Outcome and Follow‐Up)

The management of this patient required a multidisciplinary approach. The treatment regimen focused on addressing both the motor symptoms of PD and the neuropsychiatric symptoms associated with FD.

The patient's treatment regimen included Levodopa/Benserazide (250 mg every 3 h) to manage his Parkinsonian symptoms. Levodopa is a precursor of dopamine, which helps replenish the diminished levels of dopamine in the brain. Benserazide prevents the peripheral breakdown of Levodopa, ensuring more reaches the brain [13]. Amantadine (100 mg twice daily) was added to the regimen to help with motor symptoms. It acts as an NMDA receptor antagonist and increases the release of dopamine. Recently prescribed selective serotonin reuptake inhibitors (SSRIs) aimed to manage the patient's depressive symptoms. SSRIs help increase the levels of serotonin in the brain, which can improve mood and alleviate depression.

In addition to pharmacological treatments, non‐pharmacological interventions were crucial for this patient. Physical therapy was employed to address gait disturbances and rigidity. Regular exercise can improve mobility, flexibility, and overall physical function. Cognitive Behavioral Therapy (CBT) was recommended to help the patient manage his obsessive thoughts and depression. CBT can help patients manage their symptoms by changing thought patterns and behaviors. Occupational therapy was provided to help the patient regain independence in daily activities and improve his quality of life. Participation in support groups for PD and neuropsychiatric disorders was encouraged to provide emotional support and coping strategies.

Altogether, there were no special adverse or unanticipated events in the course of diagnostic and treatment procedures being reported. The patient's numbness was revealed after 1 month, with no evidence of recurrence during 6 months of the follow‐up period. Based on the patient's perspective, the therapy plan did not work for emotional, mood, and behavioral problems.

The patient underwent follow‐up evaluations at intervals of 2 to 3 months, totaling three visits. Throughout these assessments, no significant improvement or progression in clinical symptoms was observed. Due to the patient's low level of compliance, any changes in the treatment plan to achieve better outcomes were not possible.

## 5 Discussion

FD is primarily characterized by abnormal calcifications in the brain, which are often detected through neuroimaging. The pathophysiology involves disruptions in calcium and phosphate homeostasis, leading to calcium deposits in brain tissues [14]. The clinical guidelines for diagnosing Fahr's syndrome involve identifying bilateral calcification on a brain CT scan, autosomal dominant inheritance, the lack of infection, toxins, or drugs, the absence of mitochondrial dysfunction, and the presence of worsening neurological symptoms [2].

EOPD shares many features with typical PD but occurs at a younger age and often has a stronger genetic component [15]. The pathophysiology of EOPD involves the degeneration of dopaminergic neurons in the substantia nigra, a brain region critical for motor control. This degeneration leads to a decrease in dopamine levels, resulting in the motor symptoms characteristic of PD [16].

Typical signs of FD consist of movement issues similar to PD, seizures, fainting, lack of coordination, and cognitive decline [17]. On the other hand, individuals with EOPD may experience a less severe condition, but with a higher occurrence of muscle contractions and involuntary movements caused by medication, leading to a lower quality of life overall [18]. The clinical presentation of this patient was complex, with symptoms overlapping between FD and EOPD. The initial symptoms of numbness, gait disturbances, and rigidity pointed towards Parkinsonian syndrome. However, the presence of extensive neuropsychiatric symptoms, including visual hallucinations, obsessive thoughts, and depression, suggested an additional underlying condition.

The diagnostic process involved a thorough clinical evaluation, neuroimaging, and genetic testing. At present, CT is considered the most valuable diagnostic imaging technique to diagnose FD, surpassing MRI in terms of utility and effectiveness [17]. The diagnosis of FD relies on identifying brain calcification through a CT scan, along with the associated clinical symptoms, without any proven abnormalities in calcium and phosphate levels in the blood or metabolism [19].

The management of this patient required a multifaceted approach, addressing both motor and neuropsychiatric symptoms. From a therapeutic perspective, the main objective of the medical approach is to manage neuropsychiatric symptoms [6]. Removing calcium deposits from the brain without affecting calcium in bones and other tissues seems to be an unattainable goal. Additionally, although calcium is the primary mineral deposited, various other minerals are also deposited [20].

Existing literature suggests that mood stabilizers, particularly anticonvulsant medications, are associated with positive outcomes in cases where individuals exhibit psychiatric symptoms (such as lack of impulse control, behavioral abnormalities, and manic‐depressive features) or neurological symptoms (including neuromotor and neurocognitive issues) [6]. Additionally, lithium appears to offer potential therapeutic benefits in preventing and treating this neurodegenerative condition [21].

In our case, the pharmacological treatment focused on replenishing dopamine levels and alleviating Parkinsonian symptoms with Levodopa/Benserazide and Amantadine. SSRIs were used to manage depressive symptoms. Additionally, Non‐pharmacological interventions, such as physical therapy, cognitive‐behavioral therapy, and occupational therapy, played a critical role in improving the patient's overall function and quality of life. Regular physical therapy helped manage gait disturbances and rigidity, while cognitive‐behavioral therapy addressed obsessive thoughts and depression. Occupational therapy provides strategies to improve daily functioning and independence.

Given the hereditary nature of both FD and EOPD, genetic counseling was recommended for the patient and his family. Genetic counseling can provide valuable information about the risks of passing these conditions to future generations and inform family members about potential symptoms to monitor. Genetic testing provided further insights into the possible genetic causes of the patient's symptoms, identifying variants in the JAM2 and WNK1 genes.

Ongoing follow‐up and regular evaluations are essential for managing the patient's evolving clinical picture. Based on the progression of symptoms and the patient's response to therapy, adjustments to the treatment regimen may be necessary.

Here, we emphasized the potential biochemical overlap between PFBC and other neuropsychiatric diseases, as suggested by earlier research. While there is no direct evidence of a pathogenic link between FD and EOPD in the literature, our findings suggest possible correlations, including shared genetic abnormalities and environmental variables. These correlations could pave the way for new research avenues in the field of neurology and psychiatry, offering hope for further understanding and potential treatments. This detailed report also provides important information on how similar symptoms of movement disorders can result in misdiagnoses during the differential diagnosis process. It also highlights the significance of using advanced imaging methods like CT or MRI to detect basal ganglia calcifications specific to FD, which are frequently overlooked in standard diagnostic procedures for PD. By presenting a real‐life case, our study aims to enhance clinical understanding and accuracy in diagnosing uncommon neurodegenerative conditions.

There is a pressing need for further research in FD, as there is currently no therapy that can limit the progression of basal ganglia calcification [19]. Our current management and treatment strategies are primarily focused on providing symptomatic relief, closely tied to the clinical features. Unfortunately, the patient was not motivated to follow up and continue the diagnostic and treatment measures, but in any case, due to the appropriate family support, he was on the right track with the measures. We guess that one of the major reasons for this patient's poor adherence to treatment was the expense of diagnostic tests, especially genetic tests, and the long duration of the therapy plan.

## 6 Conclusion

This case underscores the importance of a comprehensive diagnostic approach in patients with overlapping neurodegenerative disorders. Symptomatic management remains crucial, with ongoing evaluations and genetic counseling recommended for the patient and his family. The co‐occurrence of EOPD and FD presents a unique clinical challenge, necessitating a multidisciplinary approach to care.

Regular neurological and neuropsychiatric evaluations, careful consideration of medication side effects, and genetic counseling for the patient and family are recommended. Additionally, further research into the genetic basis and pathophysiology of these conditions is necessary to improve diagnostic and therapeutic strategies.

## Author Contributions

**Erfan Sanaei:** project administration, writing – original draft. **Reza Bidaki:** supervision, writing – review and editing. **Mohammad Pashmchi:** investigation, writing – original draft, writing – review and editing. **Hamid Jalalifard:** data curation.

## Ethics Statement

The human studies were approved by the Ethics Committee of Shahid Sadoughi University of Medical Sciences, Yazd, Iran (Approval code: IR.SSU.MEDICINE.REC.1403.045). The local legislation and institutional requirements conducted the analyses.

## Consent

The participants provided their written informed consent to participate in this study. The patient provided written informed consent for the publication of any potentially identifiable images or data included in this article.

## Data Availability

The original contributions presented in the study are included in the article. Further inquiries can be directed to the corresponding author.
